# Elucidating the Evolutionary Relationships among *Bos taurus* Digestive Organs Using Unigene Expression Data

**DOI:** 10.4061/2009/803142

**Published:** 2010-02-08

**Authors:** D. C. Beck, Honglin Jiang, Liqing Zhang

**Affiliations:** ^1^Department of Computer Science, Virginia Polytechnic Institute and State University, 114 McBryde Hall, Blacksburg, VA 24060, USA; ^2^Department of Animal and Poultry Sciences, Virginia Polytechnic Institute and State University, Blacksburg, VA 24060, USA

## Abstract

Although the nature of ruminant evolution is still disputed, current theory based on physiology and genetic analysis suggests that the abomasum is the evolutionarily oldest stomach compartment, the rumen evolved some time after the abomasum, and the omasum is the evolutionarily youngest stomach compartment. In addition, there is some evidence of relaxed selective constraint in the stomach-like organ and the foregut shortly after the foregut formation event. Along with the assumption of a mean, stochastic rate of evolution, analysis of differences in genetic profiles among digestive body organs can give clues to the relationships among these organs. The presence of large numbers of uniquely expressed entries in the abomasum and rumen indicates either a period of relaxed selective constraint or greater evolutionary age. Additionally, differences in expression profiles indicate that the abomasum, rumen, and intestine are more closely related to each other, while the reticulum and omasum are more closely related to the rumen. Functional analysis using Gene Ontology (GO) categories also supports the proposed evolutionary relationships by identifying shared functions, such as muscle activity and development, lipid transport, and urea metabolism, between all sections of the digestive tract investigated.

## 1. Background

Domestic cattle, or *Bos taurus*, are useful organisms for genomic studies. As ruminants, they occupy an important position in the evolutionary tree and display interesting phenotypic characteristics. Additionally, domestic cattle have been bred under human-enforced selection for centuries; thus, the well-studied variations among breeds and recorded lineage provide a supplement to genetic data.

One obvious point to begin genomic analysis of domestic cattle is the digestive system. Like other ruminants, *Bos taurus* has four stomach-like compartments, as opposed to the single stomach in many other mammals. These four chambers allow digestion of structural carbohydrates contained in plants. This particular morphologic/phenotypic characteristic of ruminants and the physiological studies resulting from its prominence provide hypotheses for genetic analysis. 

The Bovine Genome Sequencing Project (BGSP) is developing a number of genetic resources for *Bos taurus* in addition to sequencing the bovine genome. One of these resources, the National Center for Biotechnology Information's (NCBI) Unigene database [1], contains data on gene expression localized to selected body sites, which mostly correspond to organs, of *Bos taurus*. This expression data, when coupled with the mean stochastic rate of mutation assumed in evolutionary genomics, provides insight on selection constraint, evolutionary age, and evolutionary relationships among these body sites. In addition, this data provides information on specific expressional differences among body sites. When linked by annotated information with Unigene data obtained from other species, this data provides insight into evolutionary relationships and specific expressional differences among the body sites of different species.

A number of physiological and genetic studies of the evolutionary relationships between the members of *Artiodactyla* have been published; however, the exact nature of these relationships is still disputed [2, 3]. Current theory maintains that *Artiodactyla* diverged into multiple groups. Of these groups, only *Ruminantia* and *Camilidae* are considered true ruminants, and they are considered to be a case of convergent evolution [2, 3]. Suborder *Ruminantia* diverged into *Pechora* and *Tragulina*; among other differences, the former has a fully developed omasum and the latter has a poorly developed, pseudo-omasum [4]. Due to physiological characteristics of ruminant digestion, physiological comparisons among members of *Ruminantia*, and genetic sequence analysis, it is believed that the abomasum is evolutionarily the oldest stomach compartment and that the omasum is evolutionarily the youngest stomach compartment [4]. Additionally, it is suggested that the stomach-like organ and the foregut underwent a period of relaxed selective constraint shortly after the evolutionary foregut formation event [5, 6].

In this study, the evolutionary relationships among the organs of the bovine digestive system were examined and compared against a set of control body sites consisting of tissues and organs. In addition, cross-species comparisons were conducted against *Homo sapiens* and *Mus musculus* using Unigene data linked by annotated information. This analysis was compared to existing physiological and genetic analyses on ruminant evolution. This analysis confirms the relative evolutionary ages of the four stomach-like organs of the bovine digestive system suggested by Langer [4]. Additionally, this analysis indicates a period of relaxed selective constraint, lends support to a potential mechanism for rumination, and elucidates some differences in gene expression among digestive organs in the three species.

## 2. Results and Discussion

The evolutionary relationships among the digestive organs of the ruminants have been previously suggested [4]. However, little evidence for these relationships has been provided at the molecular and functional level. Taking advantage of the recently developed gene expression database for *B. taurus*, the evolutionary relationships among different functional compartments, that is, organs and tissues, can be determined using gene expression profiles and the assumption of a mean, stochastic rate of evolution.

Recent work has indicated that gene expression divergence is subject to purifying selection and possibly positive selection [7]. While previous data indicates a rate of gene expression divergence that is significantly less regular than the rate of gene sequence divergence, it does not reveal any regularity in the rate of gene expression divergence for tissue-specific profiles. It is noted that an inverse relationship between gene expression intensity and protein evolution rate exists [7]. This is consistent with the previously suggested relaxed selective constraint of the digestive tract of foregut fermenters [5, 6].

Nevertheless, it is still expected that compartments which are closely related, in evolutionary terms, will express sets of genes with higher set similarity than compartments that are more distantly related. Thus, measurements of set similarity will provide some quantitative and qualitative evaluation of the evolutionary distance between functional compartments. Due to the stochastic nature of gene expression data and due to the unrecorded variation in experimental procedures used to obtain the expression data, the focus is on performing a qualitative analysis of the expression data.

### 2.1. Summary Statistics of the Unigene Data for the Three Species

Summary statistics for all available Unigene data used in this analysis are provided in Table 1. For the selected body sites, excluding the spleen, *Homo sapiens* has the largest number of Unigene entries and *B. taurus* has the fewest Unigene entries. For spleen, *Mus musculus* has the largest number of Unigene entries and *Bos taurus* has the fewest. 

However, comparison of the percentages of entries that have been annotated with Entrez identifiers indicates that *H. sapiens* has the lowest percentage of annotated entries, while *M. musculus* has the highest percentage of annotated entries. This suggests two things. First, given that *H. sapiens* is fairly wellstudied, this indicates that there are a greater number of unknown transcripts that have not been characterized at the gene level. Second, given that *M. musculus* is considered to be better studied than *H. sapiens*, this indicates that the characterization of the *M. musculus* genome has less uncertainty than that of* H. sapiens* or *B. taurus*.

Comparison of the mean expression level, using transcripts per million (TPM), indicates that, for almost all body sites, *H. sapiens* has the lowest level of expression, followed by *M. musculus* and *B. taurus*, respectively. There is also a negative correlation between number of Unigene entries in a body site and mean expression level. This indicates that in all species, and especially in *M. musculus*, that unannotated Unigene entries are expressed at low levels.

Limitations of the current Unigene database are also highlighted in these summary statistics. When compared to Unigene data for* H. sapiens* or *M. musculus*, the Unigene data for *B. taurus* has a higher percentage of entries annotated with Entrez identifiers or sequence similarity information but usually has a far lower number of entries per body site. This is indicative of an experimental bias towards gene products that are better understood and away from novel genes unique to *B. taurus*. Additionally, comparing the body sites of *B. taurus*, it is noted that some of these body sites, many of which, notably, are digestive organs, have significantly fewer entries than others. This will cause an increase in perceived distances between organs, but given the size of the samples and barring an experimental bias, this increase should be proportional in all body sites. However, in some cases, such as the omasum, the number of entries might be insufficient for any conclusion. 

Also, unknown experimental procedures and unknown normalization procedures must be considered. No information on experimental methods or on normalization procedures applied to the experimental data is included in the NCBI Unigene database. One must also consider the possibility of experimental bias via incomplete data, for example, a large number of entries from a single point in a large organ of composite structure combined with relatively few entries from other points in this organ. The intestine is an example of one of these organs; there is some evidence of this type of error discussed in the results. These caveats must be borne in mind whilst drawing any conclusions from this data.

### 2.2. Analysis of Expression Similarity and Difference between Organs

Due to stochastic noise inherent in gene expression data and unknown but potentially differing experimental procedures used in collecting this data, the data was normalized by categorizing Unigene clusters into groups based on percentiles, or “bins”, by expression level, using threshold values obtained from the distribution of expression levels in each body site. These critical values are listed in Appendix 2. Although analyses using different numbers of bins were conducted, for analysis of expression level differences, it proved sufficient to use only two bins representing expressed genes, that is, expression level greater than zero, and unexpressed genes, that is, expression level of zero.

To qualitatively evaluate the similarity between compartments in *B. taurus*, pairwise comparisons between digestive body sites and selected control body sites were made using the Jaccard index (*J*). A Jaccard value closer to 1 indicates more similarity between compared compartments; a Jaccard value closer to 0 indicates less similarity between compared compartments. The binary state, that is, expressed or unexpressed, for each gene was considered, with expressed genes having an expression level greater than 0 and unexpressed genes having an expression level of 0. 

Pairwise similarity comparisons of *B. taurus* digestive body sites, shown in Table 2, indicate that the abomasum and intestine are the most similar (*J* = 0.3492) and the omasum and intestine are least similar (*J* = 0.00793). The omasum tends to have lower similarity scores when compared to any body site, but this is at least partly due to insufficient data for that body site. The control body sites generally show higher similarity scores, with the exception of the spleen. This is counterintuitive, as it would be expected that older, more established organs would show less similarity than the newer digestive organs. However, there are more Unigene entries for the control body sites, which indicate a bias in the data collection process towards uniquely expressed entries in the digestive body sites that enhances differences between digestive body sites.

These pairwise comparisons indicate that the abomasum, rumen, and intestine are closely related, and that the reticulum is more closely related to the rumen than other organs. No conclusions can be drawn for the omasum, which is likely due to insufficient data. The raw numbers of genes expressed in each body site, portrayed in a Venn Table Appendix 1, support these conclusions and also give evidence as to the evolutionary ages of the digestive body sites. The large numbers of uniquely expressed genes in the abomasum and rumen provide evidence either to support greater evolutionary age of both organs or evidence of relaxed selective constraint on both organs [5, 6]. The large numbers of genes shared among these two organs and the intestine also give evidence for their close relationship, but do not provide any additional information due to the relatively small number of genes from the abomasum and rumen as compared to the number of genes from the intestine.

**Figure 1:**
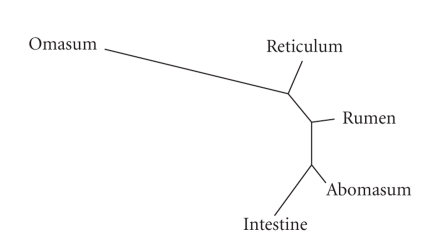
Two computed distances trees for *Bos taurus* digestive body sites using the neighbour-joining algorithm (the UPGMA tree is the same, not shown here). The distance measurements utilized during tree construction were the Jaccard distances computed in Tables 3, 4, and 5.

While the pairwise comparisons provide some evidence about the relationships among different compartments, they do not provide a complete picture. The Jaccard values were used to construct distance trees using both UPGMA and neighbor-joining algorithms. These trees, shown in Figure 1, indicate that the intestine and abomasum are most closely related, with the rumen being the next most closely related, followed by the reticulum and omasum, respectively. These results correspond to proposed evolutionary relationships among ruminant digestive organs suggested by previous physiological and genetic studies [4]. 

Pairwise comparisons of *H. sapiens* and *M. musculus*, shown in Table 3, indicate that body sites are related within a species but are also closely related to body sites of a similar type across species. Again, within each species, the intestine and stomach are most similar. The Jaccard values for *H. sapiens* stomach are curious but could indicate experimental bias in sampling, since the body site intestine as given in the Unigene database is not an accurate term in the physiological sense. The average similarity values are higher for all body site comparisons which, given the larger number of Unigene entries for *H. sapiens* and *M. musculus*, provides more evidence of experimental bias in favor of uniquely expressed entries in *B. taurus* digestive body sites.

Pairwise comparisons of the digestive body sites of *B. taurus* and the digestive body sites of *H. sapiens* and *M. musculus* are presented in Table 4. Because this part of the analysis was performed on entries that had sequence similarity annotation to the other species, this analysis utilized a fewer number of Unigene entries and is likely biased against novel genes. The trends of similarity of body sites within species and similarity by body site across species also hold for this comparison. However, *B. taurus* intestine is most similar to the intestine of *H. sapiens* and *M. musculus*, while the similarity scores of *H. sapiens* intestine and stomach are much higher than those for other body sites. The cause of this is unknown, but it could again be the result of sampling error in the Unigene data. The similarity scores for *B. taurus* reticulum and omasum are low when compared to other *B. taurus* body sites, but this may be due to insufficient data.

### 2.3. Functional Analysis of Highly Expressed Genes in Each Stomach Compartment

A number of highly expressed genes of biological relevance in each body site of *B. taurus* were identified. Some genes were unique to one body site while others were shared among body sites. Analyses using the BiNGO plug-in for the Cytoscape software package [8] were performed for each group of genes in order to find statistically enriched functions. Table 5 contains a list of GO functions corresponding to groups of highly expressed genes that are statistically significant (hypergeometric test, significance level 0.05) and are of known biological relevance.

Identification of groups of genes with biologically relevant function that are shared between *Bos taurus* digestive organs provides evidence to support an evolutionary timeline for ruminant digestive system development. As depicted in Table 5, the abomasum and the rumen both share categories of genes related to muscle development and contraction. As both the rumen and abomasum make use of contracting muscle fibers in the course of proper functioning [9], this indicates inherited function from one digestive organ to the other; it is likely that this function in the rumen was inherited from the abomasum as the abomasum-like stomach is common in other vertebrates. The reticulum and rumen share genes related to fatty acid transport and the urea cycle. This again indicates a relationship based on inherited function as the rumen and reticulum are closely related physiological function [9]. The gene ARG1 (383), which is involved in urea metabolism, is highly expressed in both the rumen and reticulum. In addition, when genes expressed in bovine digestive organs at low and medium levels were examined, the enzymes ASS (445) and ASL (435), both involved in the urea cycle, were found to be expressed in the rumen, reticulum, or intestine. The presence of a mechanism for urea metabolism is suggested by the ingestion of nitrogenous compounds from soil by ruminants. The omasum and rumen share genes involved in cross-membrane transport and in muscular activity and development. The omasum serves to absorb fluids [10], reflects particulate matter back to the rumen and absorb products of cellulose degradation, and also utilizes contracting muscle fibers in the course of proper functioning [9].

The results of the functional analysis also uncovered some results that have been noted in previous studies on bovine molecular biology. As shown in Appendix 3, Homo sapiens stomach and *Bos taurus* abomasum share the lysozyme genes LYZ3 (Entrez-Gene: 281289) and LYZ (Entrez-Gene: 781349). However, when viewing genes shared among the human stomach, bovine abomasum, and various other digestive organs that are expressed at various levels, the genes LYZ1 (Entrez-Gene: 281287) and LYZL2 (Entrez-Gene: 119180) are listed, which indicate the presence of more shared lysozyme genes [10]. It is likely that the analysis techniques used are not sensitive to detect the expected variation in expression noted in other studies [11].

## 3. Conclusions

### 3.1. Expression Analysis Reveals Results That Correspond to Current Theories on Ruminant Evolution

Analysis of the action of selective constraint on gene expression levels in the digestive system of *Bos taurus* reveals results that correspond to current theories on ruminant evolution [4]. There is evidence of relaxed selective constraint or greater evolutionary age [5, 6] in the abomasum and the rumen as indicated by the large number of genes expressed uniquely in both organs, and this analysis also indicates appropriate evolutionary ages for other digestive organs. Additionally, similarity measurements provide a sequence of evolution that corresponds to theories proposed based on previous genetic and physiological studies [4]. The abomasum and intestine are closely related, as are the stomach and intestine in nonruminants, and the rumen is closely related to the abomasum and intestine as well as the reticulum and omasum. These results, plus previous reviews that suggest that foregut evolution is evolutionarily easy [12], indicate an early foregut evolutionary event stemming from the intestine and later development of compartments stemming from a subsequently evolved rumen.

Given that the evolution of rumination is believed to have occurred separately in *Camilidae* and *Ruminantia*, and that the starting point for this development, foregut fermentation, appears to be relatively simple, this generates some interesting questions regarding the evolutionary mechanisms of complex organs and organ systems. Future work in the genomics, using camelids and true ruminants as a model for convergent evolution, might yield insight into the nature of these processes. 

### 3.2. Biological Relevant Groups of Genes Are Identified in the Digestive Organs of *Bos taurus*

Several biological relevant enriched GO clusters were noted. Some of these clusters reinforce relationships noted elsewhere in the paper. As shown in table 5, the abomasum and rumen share genes involved in muscle development and involuntary muscle activity, while the reticulum and rumen share genes involved in lipid and fatty acid transport. Given the relationships noted elsewhere, these shared genes reinforce the idea that the abomasum and rumen are evolutionarily closely related and that the reticulum and rumen are evolutionarily closely related. Additionally, the omasum and rumen share genes involved in involuntary muscle activity and signaling. These shared genes indicate both a close evolutionary relationship and confirm known physiology of the rumen and omasum. Evidence of a mechanism for urea metabolism is indicated by the expression of the ARG1 gene in the rumen and reticulum and the expression of ASS and ASL genes in other bovine digestive organs. 

The functional analysis also supported the results of previous studies of bovine molecular biology. Lysozyme genes LYZ, LYZ1, LYZ2, and LYZ3 were identified as being expressed in human and bovine digestive organs; however, the techniques used were not sensitive enough to determine differences in expression level as shown in previous studies [11]. Additional lysozyme genes LYZL4 and LYZL6 were identified but were marked as not being expressed in any digestive organs. This could be due to insufficient Unigene data.

## 4. Materials and Methods

The Unigene datasets for *Bos taurus*, *Homo sapiens*, and *Mus musculus* were downloaded from the NCBI ftp site [1] on 16 February 2008. Digestive body sites were selected by straightforward criteria. The intestine was selected for all organisms, the stomach was selected for *Homo sapiens* and *Mus musculus*, and the abomasum, omasum, reticulum, and rumen were selected for *Bos taurus*. Control body sites were of two types: tissue and composite organ. Both types were selected for ubiquity and in order to provide a representation across type and function. Selected control tissues were blood, skin, muscle, and brain. Selected control organs were liver, kidney, ovary, and spleen.

For each organism, statistical properties were computed for the Unigene entries expressed in each body site using the R statistical language. The distribution for all mean values was of a one-tailed type. The statistical values were useful for tracking certain types of error. As shown in Table 1, the numbers of data points for the omasum, reticulum, blood, and spleen in *Bos taurus* are low. In addition, the variance in mean expression values of the omasum body sites for* Bos taurus* indicates inconsistent normalization procedures, which introduces unquantifiable errors into the analysis.

To minimize errors generated by different experimental and data processing procedures, two approaches were utilized to evaluate gene expression in different body sites. The first approach used a binary criteria; if gene expression was greater than 0 transcripts per million (TPM), the gene was considered to be expressed in the body site. Otherwise, the gene was considered to be not expressed in the body site. The second approach used two computed critical values to categorize the level of gene expression into three levels: low, medium, and high. Unigene entries in the top 10 percent of expression level in any given body site were considered to be highly expressed, and entries in the bottom 75 percent of expression level were considered to be lowly expressed, with those falling in between those two values considered to be moderately expressed. The two critical values were computed independently for each body site in order to reduce the effects of inconsistent normalization. The upper and lower critical values were taken from the expression values of the elements on the 10% and 75% boundaries (see in Supplementary Material available online at doi: 10.4061/2009/803142. Table 2 that contains the critical expression values in TPM). All subsequent comparisons between body sites were performed using the values obtained from the two approaches and the results were found to be qualitatively similar. Therefore, for the sake of brevity, only the results obtained using the binary approach are presented.

The Unigene expression data was mined using custom batch files for the PostGRES SQL server and the PSQL client software. Binary Venn tables were constructed by testing all entries for expression across all selected body sites. Entries were either expressed in a body site, in which case their expression values were greater than zero, or were not expressed in a body site, in which case their expression values were equal to zero or contained no data. Pairwise similarity was computed using the standard two-set Jaccard index of similarity. The Jaccard index measures the degree of similarity between two sets, with the maximum value of 1 and minimum value of 0. Higher Jaccard indices between two body sites indicate a greater similarity between the two sets of genes that are expressed in the two body sites.

Species-species cross-linking was accomplished by joining the entries of two species, using the SQL join algorithm, through sequence similarity score annotations. If properly annotated, each Unigene entry is annotated with a sequence similarity score corresponding to a reference protein identifier and a list of sequence similarity scores corresponding to protein identifiers in other organisms. Two lists of cross-linked entries were created for each species-species cross using these annotations; one list used the organism-specific protein identifier for the first species and the reference protein identifier for the second species to link entries, while the second list used the organism-specific protein identifier for the second species and the reference protein identifier for the first species to link entries. This technique posed problems due to the poor annotation of *Bos taurus* entries and required manual analysis of the data obtained from these subsets.

In order to study the functional relationships among the genes expressed in body sites, the Cytoscape plug-in BiNGO [8] was used to perform a Gene Ontology-(GO-) based analysis on each set of genes shared among body sites or unique to a single body site. BiNGO maps a gene set of interest to the GO hierarchical graph and determines the GO terms that are statistically over- or underrepresented in the gene set using either hyper-geometric or binomial tests [8]. To minimize the influence of stochastic noise and difference in techniques among the different experiments used to generate Unigene data, functional analysis was restricted to highly expressed genes in order to get the most distinctive differences between body sites. Genes with expression values in the highest 5% of the expression values of all genes in the body site were considered to be highly expressed. This criterion is very stringent but ensures high data quality. Therefore, these results are conservative.

## Supplementary Material

Appendix 1: This file provides raw numbers of Unigene entries shared among groups of body sites within each species. Each species is presented as separate Venn tables for control and digestive body sites with each body site treated as a set of Unigene entries.
Appendix 2: This file provides a table of computed critical expression values for digestive and control body sites used in the analysis.
Appendix 3: This file provides a full listing of enriched Gene Ontology (GO) clusters for each digestive body site in *Bos taurus*.

## Figures and Tables

**Table 1:** General information about the amount and annotation levels of Unigene data used in the expression analysis of *Bos taurus*, *Homo sapiens*, and *Mus musculus*. All selected body sites for the three species are included.

Body Site		Percentage of Entries	Percentage of Entries	Mean Expression
	Number of Entries	Expressed in Body	Expressed in Body Site with	Level in Pseudo
	Expressed in Body Site	Site with Entrez	Sequence Similarity	transcripts per
		Gene Identifiers	Annotations to *H. sapiens *	Million (TPM)
Stomach (*Hs*)	15045	74.796	78.006	66.46725
Stomach (*Mm) *	8008	77.734	77.535	124.8751
Abomasum	6381	77.245	81.147	156.7153
Omasum	1275	81.098	82.902	784.314
Reticulum	3496	86.642	89.159	286.0413
Rumen	5340	83.951	86.404	187.2574
Intestine (*Bt*)	12197	76.666	80.749	81.98734
Intestine (*Hs) *	23914	61.993	66.589	41.8165
Intestine (*Mm) *	12429	82.372	81.656	80.45698
Blood (*Bt*)	1956	84.049	87.474	511.2477
Blood (*Hs*)	16169	72.268	75.849	61.846674
Blood (*Mm*)	6168	88.651	88.700	162.1272
Brain (*Bt*)	19724	62.411	67.354	50.69966
Brain (*Hs*)	39934	47.864	52.917	25.04132
Brain (*Mm*)	28605	61.328	56.777	35.63157
Muscle (*Bt*)	9506	79.960	84.957	105.1967
Muscle (*Hs*)	17176	72.438	75.215	58.22079
Muscle (*Mm*)	6891	90.001	89.755	145.1169
Skin (*Bt*)	9313	81.757	85.912	107.3768
Skin (*Hs*)	17784	73.628	77.502	56.23032
Skin (*Mm*)	15316	80.628	77.435	65.2912
Kidney (*Bt*)	10734	78.424	82.402	93.16073
Kidney (*Hs*)	25660	59.727	62.927	38.97116
Kidney (*Mm*)	14113	82.505	81.103	70.85666
Liver (*Bt*)	11520	68.941	70.920	86.80554
Liver (*Hs*)	19921	67.190	70.815	50.19828
Liver (*Mm*)	12381	85.413	85.147	80.76893
Ovary (*Bt*)	9822	77.449	82.040	101.8123
Ovary (*Hs*)	17067	69.965	73.551	58.5926
Ovary (*Mm*)	11332	83.551	81.989	88.24569
Spleen (*Bt*)	4015	81.444	84.209	249.0659
Spleen (*Hs*)	10513	81.851	84.191	95.12036
Spleen (*Mm*)	14015	79.079	75.341	71.35211

**Table 2:** Pairwise comparison of similarity among *Bos taurus* digestive and control body sites using the Jaccard index of similarity. Values closer to 1.0 denote a higher similarity in expressed genes between two body sites.

Digestive Body Sites					
	Abomasum	Intestine	Omasum	Reticulum	Rumen
Abomasum	1.0000	0.3492	0.1097	0.2300	0.2814
Intestine	0.3492	1.0000	0.0793	0.2037	0.2810
Omasum	0.1097	0.0793	1.0000	0.1343	0.1308
Reticulum	0.2300	0.2037	0.1343	1.0000	0.2641
Rumen	0.2814	0.2810	0.1308	0.2641	1.0000

Control Body Sites					
	Kidney	Liver	Ovary	Spleen	

Kidney	1.0000	0.3170	0.4031	0.2074	
Liver	0.3170	1.0000	0.3170	0.1702	
Ovary	0.4031	0.3170	1.0000	0.2320	
Spleen	0.2074	0.1702	0.2320	1.0000	

**Table 3:** Pairwise comparison of similarity among *Homo sapiens* and *Mus musculus* digestive and control body sites using the Jaccard index of similarity. Values closer to 1.0 denote a higher similarity in expressed genes between two body sites.

Combined Digestive Body Sites				
	*Hs* Intestine	*Mm* Intestine	*Hs* Stomach	*Mm* Stomach
*Hs* Intestine	1.000	0.4865	0.6926	0.3154
*Mm* Intestine	0.4865	1.000	0.4538	0.4850
*Hs* Stomach	0.6926	0.4538	1.000	0.3162
*Mm* Stomach	0.3154	0.4850	0.3162	1.000

Human Control Body Sites				
	Kidney	Liver	Ovary	Spleen

Kidney	1.000	0.4383	0.4148	0.3086
Liver	0.4383	1.000	0.4369	0.3545
Ovary	0.4148	0.4369	1.000	0.3622
Spleen	0.3086	0.3545	0.3622	1.000

Mouse Control Body Sites				
	Kidney	Liver	Ovary	Spleen

Kidney	1.000	0.4976	0.4086	0.4570
Liver	0.4976	1.000	0.3910	0.4450
Ovary	0.4086	0.3910	1.000	0.3743
Spleen	0.4570	0.4450	0.3743	1.000

**Table 4:** Pairwise comparison of similarity among *Bos taurus, Homo sapiens,* and *Mus musculus* digestive body sites using the Jaccard index of similarity. Values closer to 1.0 denote a higher similarity in expressed genes between two body sites.

Comparison Between *B. taurus* (Bt) and *H. sapiens* (Hs) Digestive Body Sites							
	Abomasum	Intestine	Omasum	Reticulum	Rumen	*Hs* Intestine	*Hs *Stomach
Abomasum	1.000	0.4707	0.1899	0.3504	0.4128	0.3049	0.3157
Intestine	0.4707	1.000	0.1400	0.3173	0.4113	0.5415	0.4804
Omasum	0.1899	0.1400	1.000	0.2299	0.2169	0.0754	0.0812
Reticulum	0.3504	0.3173	0.2299	1.000	0.3877	0.1908	0.1998
Rumen	0.4128	0.4113	0.2169	0.3877	1.000	0.2689	0.2751
*Hs* Intestine	0.3049	0.5145	0.0754	0.1908	0.2689	1.000	0.7112
*Hs* Stomach	0.3157	0.4804	0.0812	0.1998	0.2751	0.7112	1.000

Comparison Between *B. taurus* and *M. musculus* Digestive Body Sites							
	Abomasum	Intestine	Omasum	Reticulum	Rumen	*Mm* Intestine	*Mm* Stomach

Abomasum	1.000	0.4316	0.1466	0.2990	0.3660	0.3450	0.3010
Intestine	0.4316	1.000	0.1075	0.2663	0.3677	0.5166	0.3586
Omasum	0.1466	0.1075	1.000	0.1785	0.1695	0.0870	0.1003
Reticulum	0.2990	0.2663	0.1785	1.000	0.3285	0.2191	0.2199
Rumen	0.3660	0.3677	0.1695	0.3285	1.000	0.3067	0.2908
*Mm* Intestine	0.3450	0.5166	0.0870	0.2191	0.3067	1.000	0.4685
*Mm* Stomach	0.3010	0.3586	0.1003	0.2199	0.2908	0.4685	1.000

**Table 5:** Selected enriched GO categories from the functional analysis of *Bos taurus* digestive body sites. Gene groups shared between body sites with relevant biological functions provide support to an evolutionary timeline of digestive development. *BP refers to biological process, MF molecular function, and CC cellular component.

Body sites	GO function (GO category)*	*P*-value
Abomasum/Rumen	Positive regulation of dopamine metabolic process (BP)	1.24E-03
	Erythrocyte maturation (BP)	4.94E-03
	Myosin binding (MF)	3.71E-03
	Lamin filament (nuclear) (CC)	1.24E-03

Reticulum/Rumen	Fatty acid transport (BP)	2.12E-03
	Urea cycle (BP)	5.30E-03

Omasum/Rumen	Epithelial cell maturation (BP)	1.59E-03
	Negative regulation of epithelial cell proliferation (BP)	4.77E-03
	Positive regulation of striated muscle development (BP)	1.59E-03
	Positive regulation of protein catabolic Process (BP)	1.59E-03
	Embryonic heart tube development (BP)	1.59E-03
	Adult heart development (BP)	4.77E-03
	Heart looping (BP)	3.18E-03
	Skeletal muscle regeneration (BP)	1.59E-03
	Acetylcholine receptor inhibitor activity (MF)	3.18E-03
	SH3 domain binding (MF)	3.18E-03
	PDZ domain binding (MF)	4.77E-03
	Gap junction channel activity (MF)	4.77E-03
	Intermediate filament (CC)	5.27E-03
	Multivesicular body (CC)	4.77E-03
	Fascia adherens (CC)	1.59E-03
	Mitochondrial respiratory chain (CC)	4.01E-03
