# Supplementary material for: Elucidating the Evolutionary Relationships among Bos taurus Digestive Organs Using Unigene Expression Data
Source: Int J Evol Biol. 2010 Feb 8;2009:803142. doi: 10.4061/2009/803142 (PMC3042666; doi:10.4061/2009/803142)
Supplement: Supplementary file 1 — Appendix 1: This file provides raw numbers of Unigene entries shared among groups of body sites within each species. Each species is presented as separate Venn tables for control and digestive body sites with each body site treated as a set of Unigene entries. Appendix 2: This file provides a table of computed critical expression values for digestive and control body sites used in the analysis. Appendix 3: This file provides a full listing of enriched Gene Ontology (GO) clusters for each digestive body site in Bos taurus. [file 803142.f1.pdf]

Compartment	Number of Expressed Entries	Percentage of Total Entries	Number of Expressed Entries with *H. sapiens* Sequence Similarity Scores of 95% or Higher	Percentage of Total Entries
**Digestive Organs**				
Abomasum	1175	2.664	182	0.413
Omasum	112	0.254	25	0.057
Reticulum	504	1.143	69	0.156
Rumen	1030	2.335	183	0.415
Intestine	5190	11.767	1262	2.861
Abomasum – Omasum	40	0.091	11	0.025
Abomasum – Reticulum	96	0.218	21	0.048
Abomasum – Rumen	167	0.379	57	0.129
Abomasum – Intestine	1865	4.228	667	1.512
Omasum – Reticulum	9	0.02	2	0.005
Omasum – Rumen	55	0.125	18	0.041
Omasum – Intestine	127	0.288	48	0.109
Reticulum – Rumen	123	0.279	31	0.07
Reticulum – Intestine	518	1.174	172	0.39
Rumen – Intestine	1013	2.297	442	1.002
Abomasum – Omasum – Reticulum	7	0.016	2	0.005
Abomasum – Omasum – Rumen	17	0.039	6	0.014
Abomasum – Omasum – Intestine	134	0.304	61	0.138
Abomasum – Reticulum – Rumen	56	0.127	25	0.057
Abomasum – Reticulum – Intestine	435	0.986	193	0.438
Abomasum – Rumen – Intestine	987	2.238	506	1.147
Omasum – Reticulum – Rumen	30	0.068	2	0.005

Omasum – Reticulum – Intestine	26	0.059	14	0.032
Omasum – Rumen – Intestine	76	0.172	36	0.082
Reticulum – Rumen – Intestine	356	0.807	180	0.408
Abomasum – Omasum – Reticulum – Rumen	15	0.034	3	0.007
Abomasum – Omasum – Reticulum – Intestine	55	0.125	32	0.073
Abomasum – Omasum – Rumen – Intestine	149	0.338	90	0.204
Abomasum – Reticulum – Rumen – Intestine	843	1.911	530	1.201
Omasum – Rumen – Reticulum – Intestine	83	0.188	51	0.116
All Digestive Body Sites	340	0.771	242	0.549
**Control Tissues**				
Blood	229	0.519	22	0.05
Brain	9139	20.721	1498	3.396
Muscle	1140	2.585	178	0.404
Skin	1195	2.709	153	0.347
Blood – Brain	251	0.569	70	0.159
Blood – Muscle	28	0.063	6	0.014
Blood – Skin	39	0.088	7	0.016
Brain – Muscle	2426	5.5	862	1.954
Brain – Skin	2151	4.877	730	1.655
Muscle – Skin	349	0.791	89	0.202
Blood – Brain – Skin	212	0.481	94	0.213
Blood – Brain – Muscle	196	0.444	79	0.179
Blood – Skin – Muscle	18	0.041	5	0.011
Brain – Skin –Muscle	4366	9.899	2218	5.029
All Control Tissues	983	2.229	624	1.415
**Control Organs**				
Kidney	3120	7.074	682	1.546
Liver	4751	10.772	388	0.88

Ovary	2393	5.426	466	1.056
Spleen	722	1.637	124	0.281
Kidney – Liver	1210	2.743	402	0.911
Kidney – Ovary	1524	3.455	607	1.376
Kidney – Spleen	265	0.6	86	0.195
Liver – Ovary	953	2.161	310	0.703
Liver – Spleen	189	0.429	56	0.127
Ovary – Spleen	299	0.678	99	0.224
Kidney – Liver – Ovary	2346	5.319	1235	2.8
Kidney – Liver Spleen	233	0.528	97	0.22
Kidney – Ovary - Spleen	469	1.063	219	0.497
Liver – Ovary – Spleen	271	0.614	94	0.213
All Control Organs	1567	3.552	975	2.221
