# Supplementary material for: Elucidating the Evolutionary Relationships among Bos taurus Digestive Organs Using Unigene Expression Data
Source: Int J Evol Biol. 2010 Feb 8;2009:803142. doi: 10.4061/2009/803142 (PMC3042666; doi:10.4061/2009/803142)
Supplement: Supplementary file 2 [file 803142.f2.pdf]

**Body Site**	***Bos taurus***		***Homo sapiens***		***Mus musculus***	
Stomach	N/A	N/A	61.7659	133.826	98.1868	229.103
Abomasum	173.198	346.395	N/A	N/A	N/A	N/A
Omasum	453.104	1359.31	N/A	N/A	N/A	N/A
Reticulum	151.416	302.831	N/A	N/A	N/A	N/A
Rumen	91.1245	250.592	N/A	N/A	N/A	N/A
Intestine	79.0551	173.921	38.2456	93.4893	58.2262	151.388
Blood	471.198	1178	56.397	128.908	174.257	290.428
Brain	47.0541	105.872	17.2068	56.1486	35.8559	90.6943
Muscle	69.3217	138.643	46.2317	101.71	145.492	254.61
Skin	85.1837	170.367	52.1584	123.284	67.403	143.231
Kidney	65.3603	169.937	37.6345	84.6776	64.4766	137.013
Liver	69.5543	156.497	38.4049	91.2115	54.3021	126.705
Ovary	85.1046	170.209	58.4533	116.907	72.9514	164.141
Spleen	288.184	504.323	73.996	166.491	73.6548	157.832
