# Supplementary material for: Elucidating the Evolutionary Relationships among Bos taurus Digestive Organs Using Unigene Expression Data
Source: Int J Evol Biol. 2010 Feb 8;2009:803142. doi: 10.4061/2009/803142 (PMC3042666; doi:10.4061/2009/803142)
Supplement: Supplementary file 3 [file 803142.f3.pdf]

Abomasum

Biological Process

Cellular metabolic process	51	7.96E-04
Actin filament bundle formation	2	3.97E-04
Regulation of cell shape	3	3.99E-04
Digestion	3	7.62E-04
**Molecular Function**		
Coenzyme binding	7	
Catalytic activity	64	9.28E-07
ATPase, coupled to transmembrane movement of substances	6	1.00E-03
**Cellular Component**		
Vesicle	8	6.57E-04
Golgi apparatus	9	1.41E-03
Mitochondrion	18	2.68E-05
Clathrin coat	3	1.50E-03

The abomasum is easy to understand. Proteins involved in transmembrane movement and protein creation, i.e., mitochondrial proteins, are highly expressed. This corresponds to production and secretion of digestive enzymes

Intestine

Cluster Size Too Large

Omasum

Insufficient Cluster Size

Reticulum

Biological Process

Oxygen transport	2	4.10E-03
Purine ribonucleotide transport	1	8.14E-03
Reverse cholesterol transport	1	8.13E-03
Negative regulation of cytokine secretion during immune response	1	8.14E-03
Negative regulation of lipoprotein metabolic process	1	8.14E-03
Tissue remodeling	2	1.34E-03
Ovulation from ovarian follicle	1	8.14E-03
Negative regulation of bone mineralization	1	8.14E-03
Negative regulation of blood coagulation	2	6.45E-04
Acute-phase response	4	3.86E-06
Platelet activation	3	1.02E-05
Hemolysis by symbiont of host red blood cells	1	8.14E-03
Hormone metabolic process	3	1.81E-03
Tyrosine catabolic process	2	1.95E-04
L-phenylalanine catabolic process	3	5.15E-06
Tryptophan catabolic process to kynurenine	1	8.14E-03
Heme biosynthetic process	2	6.45E-04
Protein polymerization	3	1.47E-03

Maintenance of mitochondrion location	1	8.14E-03
**Molecular Function**		
Plasmin activity	1	8.14E-03
Thrombin activity	1	8.14E-03
Protein C (activated) activity	1	8.14E-03
5-aminolevulinate synthase activity	1	8.14E-03
Betaine-homocysteine S-methyltransferase activity	1	8.14E-03
Guanidinoacetate N-methyltransferase activity	1	8.14E-03
Tyrosine transaminase activity	1	8.14E-03
Estrone sulfotransferase activity	1	8.14E-03
Hydroxymethylbilane synthase activity	1	8.14E-03
Methionine adenosyltransferase activity	1	8.14E-03
Tryptophan 2,3-dioxygenase activity	1	8.14E-03
4-hydroxyphenylpyruvate dioxygenase activity	1	8.14E-03
Phenylalanine 4-monooxygenase activity	1	8.14E-03
Chitin binding	1	8.14E-03
Oxygen binding	2	4.82E-03
Steroid binding	3	1.63E-03
Amino acid binding	2	1.33E-03
Heparin binding	4	7.24E-05
Identical protein binding	4	3.87E-03
Protein binding, bridging	3	8.21E-05
IgA binding	1	8.14E-03
Insulin-like growth factor binding	2	5.59E-03
Retinoid binding	2	3.43E-03
Iron ion binding	7	1.07E-03
Calcium ion binding	10	6.91E-03
Pyridoxal phosphate binding	3	6.53E-03
Serine-type endopeptidase inhibitor activity	11	2.79E-11
Cysteine protease inhibitor activity	5	2.52E-07
**Cellular Component**		
Fibrinogen complex	3	5.22E-07
Hemoglobin complex	2	2.83E-03

It's difficult to determine what the individual proteins do, but there are a lot involved in lipid transport and there are also a large number involved in immune response. One of the reticulum's functions is to absorb lipids, and it also acts as a trap for foreign objects. It also might have some sort of filtering mechanism for removing rumen bacteria from the digestive system.

Rumen

**Biological Process**		
Response to oxidative stress	4	8.70E-04
Defense response to bacterium	3	1.20E-02
Regulation of the force of heart contraction	2	3.62E-03
Regulation of systemic arterial blood pressure by ischemic conditions	1	1.17E-02
Ventricular cardiac muscle morphogenesis	2	4.03E-04

Atrial cardiac muscle morphogenesis	1	1.17E-02
Striated muscle contraction	2	3.62E-03
Erythrocyte differentiation	2	8.27E-03
Visceral muscle development	1	1.17E-02
Cardiac muscle development	2	7.99E-04
Muscle filament sliding	1	1.17E-02
Myofibril assembly	2	1.32E-03
Fumarate metabolic process	1	1.17E-02
Phosphocreatine biosynthetic process	1	1.17E-02
Phosphatidylcholine biosynthetic process	1	1.17E-02
Iron-sulfur cluster assembly	2	7.99E-04
ATP synthesis coupled proton transport	6	3.34E-07
Gas transport	2	1.12E-02
Mitochondrial electron transport, ubiquinol to cytochrome c	2	7.99E-04
Mitochondrial electron transport, NADH to ubiquinone	3	1.24E-04
Protein homotetramerization	2	7.99E-04
**Molecular Function**		
Microfilament motor activity	1	1.17E-02
Electron carrier activity	7	3.63E-03
Troponin C binding	1	1.17E-02
Troponin T binding	1	1.17E-02
4 iron, 4 sulfur cluster binding	2	1.12E-02
FATZ 1 binding	1	1.17E-02
ZASP binding	1	1.17E-02
Electron-transferring-flavoprotein dehydrogenase activity	1	1.17E-02
2 iron, 2 sulfur cluster binding	2	8.27E-03
Acyl carrier activity	1	1.17E-02
Cytochrome-c oxidase activity	3	1.77E-03
Ubiquinol-cytochrome-c reductase activity	2	1.97E-03
Hydrogen ion transporting ATP synthase activity, rotational mechanism	7	4.30E-07
Hydrogen ion transporting ATPase activity, rotational mechanism	6	3.30E-06
NADH dehydrogenase (ubiquinone) activity	9	7.99E-13
Iron ion binding	8	2.08E-03
Fumarate hydratase activity	1	1.17E-02
Calcium channel inhibitor activity	1	1.17E-02
Calpain inhibitor activity	1	1.17E-02
**Cellular Component**		
Adherens junction	2	3.62E-03
Proton-transporting ATP synthase complex, coupling factor F(o)	6	1.97E-09
Myosin complex	4	1.41E-03
Mitochondrial respiratory chain complex I	2	5.73E-03
Intermediate filament	5	1.24E-03
Z disc	2	2.73E-03
Mitochondrial large ribosomal subunit	2	6.94E-03

Almost all of these entries are involved with muscle activity.
It makes you wonder about bias in the collected data.

Abomasum - Intestine

Cluster Size Too Large

Rumen - Intestine

Cluster Size Too Large

Reticulum - Intestine

Cluster Size Too Large

Omasum - Intestine

Insufficient Cluster Size

Abomasum - Rumen

**Biological Process**		
Positive regulation of dopamine metabolic process	1	1.24E-03
Signal peptide processing	1	7.41E-03
Purine ribonucleoside salvage	1	4.94E-03
Erythrocyte maturation	1	4.94E-03
Hypoxanthine metabolic process	1	1.24E-03
Mature ribosome assembly	1	1.24E-03
Protein homotetramerization	1	4.94E-03
**Molecular Function**		
Ribosome binding	1	2.48E-03
Coenzyme binding	2	7.63E-03
Myosin binding	1	3.71E-03
Aspartate transaminase activity	1	3.71E-03
Hypoxanthine phosphoribosyltransferase activity	1	4.94E-03
Hydroxymethylglutaryl-CoA synthase activity	1	3.71E-03
Flavin reductase activity	1	1.24E-03
Butyryl-CoA dehydrogenase activity	1	1.24E-03
Biliverdin reductase activity	1	1.24E-03
Hydrogen-exporting ATPase activity, phosphorylative mechanism	1	8.64E-03
Metalloendopeptidase inhibitor activity	1	7.41E-03
**Cellular Component**		
Lamin filament (nuclear)	1	1.24E-03
Vacuolar proton-transporting V-type ATPase complex	1	3.71E-03
Mitochondrial matrix	2	5.02E-03
Signal peptidase complex	1	3.71E-03

There are a couple of interesting entries in this set. First, the dopamine entry is interesting because both the abomasum and rumen undergo muscular contractions as part of the digestive process.
Second, transport-related elements are noted in the Cellular Component section. Some of these specific proteins might be important, but it is hard (for me) to tell their

Reticulum - Rumen

**Biological Process**		
Fatty acid transport	1	2.12E-03
Response to ethanol	1	4.24E-03
Arginine metabolic process	1	5.30E-03
Urea cycle	1	5.30E-03
**Molecular Function**		
TATA-binding protein binding	1	1.06E-03
Arginase activity	1	2.12E-03
Aspartate transaminase activity	1	3.18E-03
Prostaglandin-F synthase activity	1	1.06E-03
**Cellular Component**		
Rough endoplasmic reticulum membrane	1	4.24E-03

The urea cycle is interesting because this has been noted in the literature. Likewise, the fatty acid transport and rough ER membrane entries are interesting because these organs are believed to absorb fatty acid byproducts from the bacterial action.

Omasum - Rumen

**Biological Process**		
ATP transport	1	1.59E-03
Response to pH	1	3.18E-03
Mitochondrial electron transport, ubiquinol to cytochrome c	1	6.35E-03
Translation	8	1.76E-03
Epithelial cell maturation	1	1.59E-03
Negative regulation of epithelial cell proliferation	1	4.77E-03
Positive regulation of striated muscle development	1	1.59E-03
Positive regulation of protein catabolic process	1	1.59E-03
Embryonic heart tube development	1	1.59E-03
Adult heart development	1	4.77E-03
Heart looping	1	3.18E-03
Skeletal muscle regeneration	1	1.59E-03
Pathogenesis	1	1.59E-03
**Molecular Function**		
Structural constituent of ribosome	7	3.79E-08
Acetylcholine receptor inhibitor activity	1	3.18E-03
tRNA binding	1	1.27E-02
Phosphopyruvate hydratase activity	1	3.18E-03
SH3 domain binding	1	3.18E-03
PDZ domain binding	1	4.77E-03
Gap junction channel activity	1	4.77E-03
Ubiquinol-cytochrome-c reductase activity	1	9.52E-03
**Cellular Component**		
Phosphopyruvate hydratase complex	1	3.18E-03
Ribosome	7	8.26E-08

Intermediate filament	2	5.27E-03
Multivesicular body	1	4.77E-03
Fascia adherens	1	1.59E-03
Mitochondrial respiratory chain	2	4.01E-03

This is very interesting. Almost all of these clusters have to do with either the lining of the organ or muscular contractions. There isn't any evidence in the literature (that I read, at least) to support the omasum having muscular contractions similar to the rumen. This is either evidence of unknown function or evidence that the rumen and omasum are closely related, evolutionarily. Very good results.

Abomasum - Reticulum

Insufficient Cluster Size

Omasum - Reticulum

Insufficient Cluster Size

Abomasum - Omasum

Insufficient Cluster Size

Cow Abomasum - Human Stomach

**Biological Process**		
Digestion	3	1.48E-05
Cell wall catabolic process	2	1.09E-03
Actin filament bundle formation	2	2.79E-05
Cytolysis	2	6.03E-04
**Molecular Function**		
Lysozyme activity	2	7.11E-04
Actin filament binding	2	6.03E-04
**Cellular Component**		
Cytoplasm	20	8.37E-06

Many of the same enriched clusters are shared across the bovine abomasum and human stomach. Those which differ are probably due to fragmentary data and annotation.
